# Association between History of Dental Amalgam Fillings and Risk of Parkinson’s Disease: A Population-Based Retrospective Cohort Study in Taiwan

**DOI:** 10.1371/journal.pone.0166552

**Published:** 2016-12-01

**Authors:** Yung-Chuang Hsu, Cheng-Wei Chang, Hsin-Lin Lee, Chuan-Chung Chuang, Hsien-Chung Chiu, Wan-Yun Li, Jorng-Tzong Horng, Earl Fu

**Affiliations:** 1 Department of Dentistry, Tri-Service General Hospital and National Defense Medical Center, Taipei, Taiwan; 2 Department of Information Management, Hsing Wu University, New Taipei City, Taiwan; 3 Department of Computer Science and Information Engineering, National Central University, Chungli, Taiwan; 4 Population-Health and Clinical Informatics Research Group, Department of Biomedical Informatics, Asia University Taiwan, Taichung, Taiwan; Kaohsiung Medical University Hospital, TAIWAN

## Abstract

The impact of dental amalgam on the development of Parkinson’s disease (PD) is still uncertain, although a positive association between dental amalgam and PD has been found in a few case-control studies. The patients with amalgam fillings restored between 2000 and 2008 were identified by using the National Health Insurance Research Database (NHIRD) in Taiwan. The same number of patients who had no new amalgam filling restored was matched by sex, age, and treatment date. Both cohorts were followed up from the treatment date until the date of diagnosis of PD, death, or the end of the year 2008. The individuals who received amalgam fillings had a significantly higher risk of PD afterward (adjusted hazard ratio [HR]=1.583, 95% confidence interval [CI]=1.122–2.234, *p*=0.0089) than those who did not. In the individuals who received amalgam fillings, being diagnosed with diabetes or hyperlipidemia demonstrated a significantly lower HR of PD occurrence than in the patients without diabetes or hyperlipidemia (HR=0.449, 95% CI=0.254–0.794, *p*=0.0059; HR=0.445, 95% CI=0.260–0.763, *p*=0.0032) after adjusting for comorbidities and Charlson-Deyo Comorbidity Index (CCI) scores. Meanwhile, hypertension increased the hazard risk of PD (HR=1.645, 95% CI=1.098–2.464, *p*=0.0159). The patients exposed to dental amalgam fillings were 1.583 times more likely to have PD afterward compared to their non-exposed counterparts after adjusting for comorbidities and CCI scores.

## Introduction

Dental amalgam, which contains high amounts of mercury, has been commonly used as a filling material for dental treatments, such as cavity restorations, endodontic retrograde root fillings, and core fabrication. Compared with other dental filling materials, amalgam is believed to have the advantages of being cost effective, sustainable, and resistant to chewing force [1], besides being easy to handle during operations. Therefore, dental amalgam is still currently one of the most commonly used posterior teeth restoration material [2]. However, the use of amalgam in dentistry has been controversial since the 19th century. Studies have shown that dental amalgam filling constantly releases mercury vapor, which might lead to degeneration of the neurological system [3, 4]. Other studies have shown that the numbers of cavity faces and teeth restored with amalgam filling have positive correlations with the mercury level found in patients’ blood and urine [5–7]. From the other point of view, the Scientific Committee on Emerging and Newly Identified Health Risks of the European Commission declared in 2015 that the evidence of the adverse effects of dental amalgam is weak and does not preclude the use of either amalgam or alternative materials in dental restorative treatment [8]. In 2015, the American Food and Drug Administration also stated that dental amalgam is safe to use in patients aged >6 years and without allergy to mercury or other metal contained in dental amalgam. However, amalgam restoration in pregnant women and children under neurological development remained a concern [9].

Parkinson’s disease (PD) is a common neurodegenerative disease characterized by neuronal cell loss in the substantia nigra and subsequently reduced secretion of dopamine. Aging is considered to be a major risk factor for PD [10], and other potential risk factors such as head injury, infection, neurotoxin, gene expression, and environmental exposure to heavy metals (e.g., mercury) have been reported [11, 12]. It has been shown that the major sources of elemental mercury vapor (Hg^0^) exposure are occupational and dental amalgam [13]. The common PD-like symptoms observed after occupational exposure to Hg^0^ include decreased strength and coordination, and increased tremor [14]. However, the impact of dental amalgam on the development of PD is still not well understood. Therefore, this study was conducted by using the National Health Insurance Research Database (NHIRD) in Taiwan (http://nhird.nhri.org.tw/en/index.htm) to evaluate whether patients aged ≥55 years who have dental amalgam filling(s) have an increased risk of acquiring PD afterward.

## Materials and Methods

### Data sources

The cohorts were selected from among patients registered in the NHIRD, which was released for research purposes by the National Health Research Institutes (NHRI) in 2008. As of 2007, 98.4% of Taiwan’s population (approximately 22.96 million) was enrolled in the NHIRD. The data used in the present study were retrieved from a dataset of 1 million randomly sampled enrollees in the mother NHIRD. This consisted of 1 million randomly selected subjects who represent about 4.5% of the Taiwanese population from the entire NHI enrollee profile. No significant differences in age and sex were found between the 1 million randomly sampled dataset and the enrollees in the mother NHIRD [15]. Patient demographic characteristics included encrypted identification numbers, sex, dates of birth and death, and diagnostic data and procedures. The diagnostic data included the dates of dental procedures and the *International Classification of Diseases*, *Ninth Revision*, *Clinical Modification* (*ICD-9-CM*) diagnostic and procedure codes [16].

### Case selection and definition

**Fig 1:**
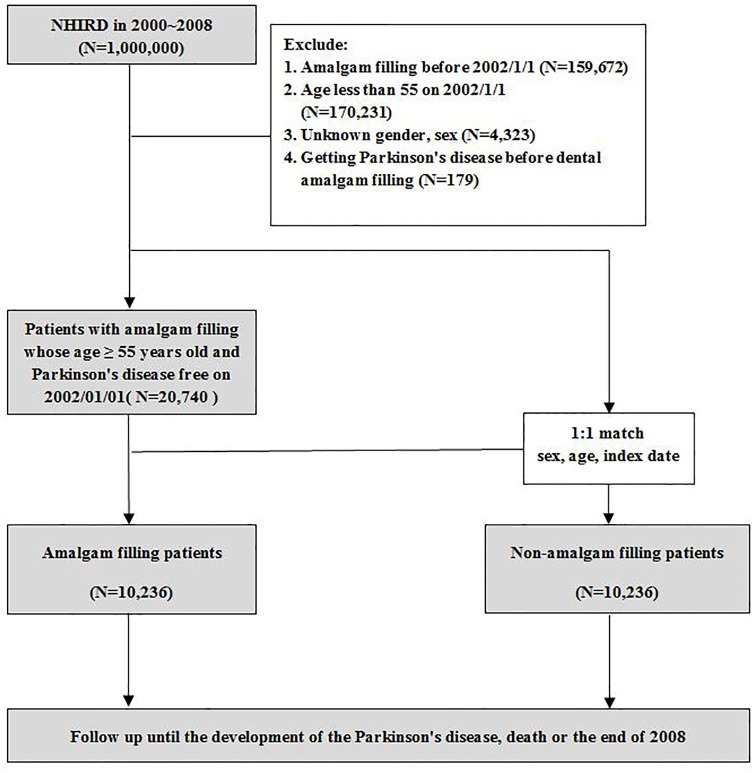
Amalgam filling cohort and non-amalgam filling cohort selection flowchart.

The cases with amalgam fillings included in this study were patients with at least one procedure code record of amalgam filling (89001C–89003C and 89101C–89103C) between 2000 and 2008. The PD cases comprised patients who were diagnosed as having PD (*ICD-9-CM* code 3320) at least once. To improve the diagnostic accuracy and exclude secondary Parkinsonism, patients who had a diagnosis of dementia, cerebrovascular diseases, head injury, or psychotic disorders at the time of or 1 year before the PD diagnosis were excluded [17]. From the 1 million representative samples from the NHIRD during 2000–2008, we excluded patients whose dates of amalgam fillings (i.e., index date) before January 1, 2002 and patients with newly diagnosed PD before the index date (Fig 1). The above exclusion process for this 2-year buffer period (January 1, 2000–December 31, 2001) can ensure that the patients with amalgam filling and PD included in this study were newfound cases during 2000–2008. Then we excluded patients aged <55 years and patients with unknown sex or age as of January 1, 2002. The previous finding has revealed that the prevalence of PD in Taiwan increased with age and steeply increased after 60 years of age [17]. Therefore, patients aged ≥55 years were included because the 7-year observation period (January 1, 2002–December 31, 2008) could cover the higher occurrence age of PD (i.e., 60 years old) in order to reduce the amount of data being collected. A total of 20,740 patients met the inclusion criteria for our study. For the non-amalgam filling cohort, we used a 1:1 paired matching for each amalgam filling patient by sex, age, and index date. We identified the amalgam filling cohort (n = 10,236) and the non-amalgam filling cohort (n = 10,236) for the data analysis. Both cohorts were followed from the index date until the PD diagnosis, death, or the end of the year 2008, whichever came first. The PD-related comorbidities [18, 19], including diabetes (*ICD-9-CM* code 250), hypertension (*ICD-9-CM* codes 401–405), hyperlipidemia (*ICD-9-CM* code 272) and cardiovascular disease (*ICD-9-CM* codes 410–414 and 425–429) were recorded if diagnosed 2 times or more. The aforementioned diseases were selected as the comorbidities because they are related to metabolic syndrome [20], which may promote neurodegenerative diseases [21]. The established Charlson-Deyo Comorbidity Index (CCI), which contains 17 weighted comorbidities and is able to predict the subsequent 1-year mortality among inpatients, was also calculated for each participant [22, 23].

### Statistical analysis

Demographic characteristics and baseline comorbidities were compared between the amalgam and non-amalgam filling cohorts. McNemar’s tests for categorical variables and paired samples t-test for continuous variables were used. The categorical variables such as age group, sex, comorbidities, follow-up group, and CCI group were reported as percentages. Continuous variables such as age, follow-up period, and CCI score are reported as mean and standard deviation. The incidence rate (per 1000 person-years) was calculated by dividing the number of events of current PD by the person-years observed for each patient. The Kaplan-Meier method was used to assess the survival probability in PD between the amalgam and non-amalgam filling cohorts. The log-rank test was used to compare their differences. Univariate and multivariable models were then used to calculate the hazard ratios (HRs) and 95% confidence intervals (CIs) with the Cox proportional-hazards regression models. Multivariable models were adjusted for age; comorbidities of diabetes, hypertension and hyperlipidemia; and moderate CCI. All the analyses were conducted by using SAS version 9.4 (SAS Institute Inc., Cary, NC, USA). Statistical significance was defined when the p-value was <0.05.

## Results

### Demographic characteristics

The demographic characteristics and comorbidities of the amalgam filling cohort (n = 10,236) and non-amalgam filling cohort (n = 10,236) are shown in Table 1. The mean age was 64.39±7.30 years in the amalgam filling cohort and 64.29±7.52 years in the non-amalgam filling cohort. The numbers of male and female patients were the same in both cohorts. The patients with amalgam fillings had a higher prevalence of all 4 comorbidities and mean CCI scores compared to the non-amalgam filling cohort (*p*<0.001). The mean follow-up time for the amalgam and non-amalgam filling cohorts was 5.15 and 5.22 years, respectively.

**Table 1:** Demographic characteristics of amalgam filling and non-amalgam filling patients.

Descriptor	Amalgam filling				P value
	Yes (n = 10236)		No (n = 10236)		
	n	(%)	n	(%)	
**Age (years) mean ± SD**	64.39±7.30		64.29±7.52		1.0000
**Age group (years)**					
55–64	6031	58.92	6031	58.92	
65–74	3044	29.74	3044	29.74	
≥ 75	1161	11.34	1161	11.34	
**Sex**					
Female	4173	40.77	4173	40.77	1.0000
Male	6063	59.23	6063	59.23	
**Comorbidities**					
Diabetes	1813	17.71	506	4.94	<.0001
Hypertension	4431	43.29	1402	13.70	<.0001
Hyperlipidemia	2039	19.92	358	3.50	<.0001
Cardiovascular disease	2679	26.17	675	6.59	<.0001
**Follow up year mean ± SD**	5.15±1.80		5.22±1.64		0.3058
**Follow up year**					
≤1	618	6.04	598	5.84	
1–4	1234	12.06	1219	11.91	
≥4	8384	81.91	8419	82.25	
**CCI score \Mean**	1.06±1.40		0.28±0.72		<.0001
CCI LOW(0)	4869	47.57	8387	81.94	
CCI Moderate(1)	2509	24.51	1218	11.90	
CCI High(≥2)	2858	27.92	631	6.16	

Definition of abbreviations: CCI = Charlson-Deyo Comorbidity Index

### Risk of PD in the amalgam filling cohort

The crude numbers of individuals with PD in the amalgam and non-amalgam filling cohorts were 126 and 56, respectively. The overall incidence of PD was significantly higher in the amalgam filling cohort than in the non-amalgam filling cohort (2.35 vs. 1.04 per 1000 person-years), with an adjusted HR (aHR) of 1.583 (95% CI = 1.122–2.234, *p* = 0.0089; Table 2). The significantly higher incidence of PD in the amalgam filling cohort was found in the 65- to 74-year age group but not in the 55- to 64-year age and ≥75-year age groups. For the patients who had no diabetes, the incidence of PD (HR = 1.680, 95% CI = 1.171–2.410, *p*<0.01) was also significantly higher in the amalgam filling cohort than in the non-amalgam filling cohort. Similar results were found in the patients without hypertension, hyperlipidemia, cardiovascular disease, follow-up of at least 4 years, and moderate CCI score. However, if the patients had PD-related comorbidities, follow-up <4 years, and low or high CCI scores, the incidence of PD would be statistically similar in the amalgam and non-amalgam filling cohorts.

**Table 2:** Incidence and hazard ratios of PD for amalgam filling patients compared with non-amalgam filling patients by demographic characteristics and comorbidities.

Variables	Amalgam filling						Crude HR (95% CI)	P value	Adjusted HR(95% CI)	P value
	Yes			No						
	Event	Person-year	Rate	Event	Person-year	Rate				
**All**	126	53526.34	2.35	56	53725.90	1.04	2.255 (1.646–3.089)	<.0001	1.583 (1.122–2.234)	0.0089
**Sex**										
Female	52	23085.52	2.25	24	23175.99	1.04	2.172 (1.339–3.522)	0.0017	1.615 (0.956–2.717)	0.0729
Male	74	30440.82	2.43	32	30549.91	1.05	2.317 (1.531–3.508)	<.0001	1.556 (0.986–2.455)	0.0577
**Age**										
55–64	41	30741.35	1.33	17	30807.55	0.55	2.412 (1.371–4.246)	0.0023	1.664 (0.903–3.064)	0.1025
65–74	59	16572.36	3.56	24	16670.93	1.44	2.468 (1.536–3.966)	0.0002	2.036 (1.208–3.430)	0.0076
≥ 75	26	6212.625	4.19	15	6247.425	2.40	1.740 (0.921–3.284)	0.0877	1.203 (0.581–2.487)	0.6188
**Diabetes**										
NO	105	44297.96	2.37	52	50863.92	1.02	2.320 (1.264–3.235)	<.0001	1.680 (1.171–2.410)	0.0048
Yes	21	9228.384	2.28	4	2861.981	1.40	1.557 (0.534–4.538)	0.4169	1.041 (0.347–3.123)	0.9422
**Hypertension**										
NO	46	30835.14	1.49	39	45789.33	0.85	1.761 (1.149–2.698)	0.0093	1.668 (1.063–2.618)	0.0261
Yes	80	22691.2	3.53	17	7936.573	2.14	1.601 (0.948–2.703)	0.0782	1.505 (0.880–2.573)	0.1354
**Hyperlipidemia**										
NO	103	43435.46	2.37	56	51649.35	1.08	2.192 (1.583–3.035)	<.0001	1.500 (1.055–2.135)	0.0241
Yes	23	10090.88	2.28	0	2076.553	0.00	N/A	N/A	N/A	N/A
**Cardiovascular disease**										
NO	77	39961.91	1.93	49	49753.11	0.98	1.962 (1.371–2.806)	0.0002	1.590(1.083–2.335)	0.0180
Yes	49	13564.43	3.61	7	3972.786	1.76	1.970 (0.892–4.352)	0.0937	1.652 (0.736–3.709)	0.2236
**Follow up year**										
≤1	40	677.0685	59.08	17	665.3479	25.55	2.301 (1.304–4.058)	0.0040	1.471 (0.736–2.940)	0.2750
1–4	40	3893.874	10.27	20	3861.74	5.18	1.988 (1.162–3.400)	0.0121	1.211 (0.636–2.307)	0.5603
≥ 4	46	48955.40	0.94	19	49198.81	0.39	2.427 (1.422–4.142)	0.0011	1.929 (1.086–3.425)	0.0249
**CCI score \Mean**										
CCI LOW(0)	43	26408.01	1.63	42	43371.51	0.97	1.697 (1.109–2.597)	0.0148	1.351 (0.857–2.129)	0.1951
CCI Moderate(1)	40	12892.46	3.10	6	6745.978	0.89	3.430 (1.454–8.090)	0.0049	3.369 (1.417–8.011)	0.0060
CCI High(≥ 2)	43	14225.87	3.02	8	3608.414	2.22	1.302 (0.612–2.771)	0.4932	1.121 (0.519–2.418)	0.7712

Definition of abbreviations: HR = hazard ratio; CI = confidence interval; PY = person-years; Rate = Per 1,000 person-years; Event = Number of Parkinson's disease; Model was adjusted for comorbidities (Diabetes, Hypertension, Hyperlipidemia, and Cardiovascular disease) and CCI score; N/A = Not Applicable

Among the amalgam filling cohort, no significant difference in hazard risk of PD was found between the male and female patients in the univariate and multivariable analyses (*p* = 0.7273 and *p* = 0.8477, respectively; Table 3). The patients aged 65–74 years (HR = 2.255, 95% CI = 1.500–3.390, *p*<0.0001) and ≥75 years (HR = 2.186, 95% CI = 1.299–3.681, *p* = 0.0032) were found to have a higher hazard risk of PD than those aged 55–64 years. The patients with diabetes (HR = 0.449, 95% CI = 0.254–0.794, *p* = 0.0059) or hyperlipidemia (HR = 0.445, 95% CI = 0.260–0.763, *p* = 0.0032) had a lower hazard risk of PD than the non-diabetic or non-hyperlipidemia patients after adjusting for comorbidities and CCI score. Meanwhile, the patients with hypertension had a higher hazard risk of PD than the non-hypertension patients (HR = 1.645, 95% CI = 1.098–2.464, *p* = 0.0159). The patients with moderate CCI scores also had higher risk of PD compared to the patients with low CCI scores (HR = 1.893, 95% CI = 1.203–2.978, *p* = 0.0058).

**Table 3:** Univariate and multivariable hazard ratios of covariates for all-cause PD among amalgam filling patients.

Variables	Univariate analysis		Multivariable analysis	
	HR (95% CI)	p-value	HR (95% CI)	p-value
**Sex** (Male as the baseline)	0.939 (0.658–1.339)	0.7273	0.966 (0.676–1.379)	0.8477
**Age**				
55–64	1		1	
65–74	2.681 (1.800–3.994)	<.0001	2.255 (1.500–3.390)	<.0001
≥ 75	3.155 (1.930–5.157)	<.0001	2.186 (1.299–3.681)	0.0032
**Comorbidities**				
Diabetes	0.580 (0.331–1.014)	0.0559	0.449 (0.254–0.794)	0.0059
Hypertension	1.751 (1.206–2.543)	0.0033	1.645 (1.098–2.464)	0.0159
Hyperlipidemia	0.581 (0.343–0.982)	0.0426	0.445 (0.260–0.763)	0.0032
Cardiovascular disease	1.204 (0.811–1.789)	0.3571	0.871 (0.569–1.335)	0.5273
**CCI score \Mean**				
CCI LOW(0)	1		1	
CCI Moderate(1)	1.896 (1.233–2.917)	0.0036	1.893 (1.203–2.978)	0.0058
CCI High(≥2)	1.831 (1.199–2.794)	0.0051	1.197 (0.936–1.532)	0.1526

### Kaplan-Meier PD-free survival curve

**Fig 2:**
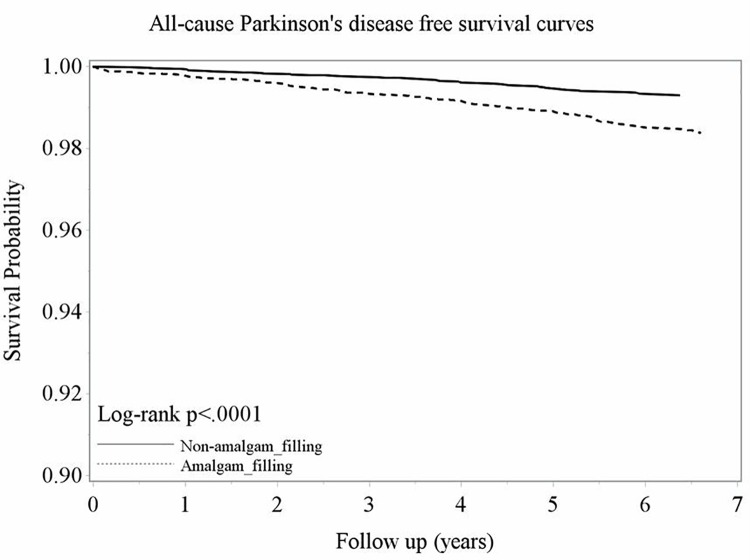
Kaplan-Meier model for estimating the PD-free survival probability of amalgam filling and non-amalgam filling patients.

The probability of PD-free survival in the non-amalgam filling cohort was higher than that in the amalgam filling cohort from 2002 to 2008 (log-rank test, *p*<0.0001; Fig 2).

## Discussion

To the best of our knowledge, this is the first nationwide population-based cohort study to evaluate the risk of developing PD after dental amalgam filling. In our study, the risk of PD increased in the patients with amalgam fillings restored when compared with the non-amalgam filling cohort regardless of sex or age (Table 2) after adjusting for the comorbidities (diabetes, hypertension, hyperlipidemia, and cardiovascular disease) and CCI scores. With the Kaplan-Meier model (Fig 2), we also demonstrated that the probability of PD-free survival was significantly lower in the patients who received dental amalgam filling than in non-dental amalgam filling (*p*<0.0001). The association between amalgam filling and the development of PD has been examined in previous case-control or cohort studies (Table 4) [4, 7, 24]. Ngim and Devathasan reported a clear monotonic dose-response association between PD and blood mercury levels in Singapore [24]. Seidler *et al*. also reported that patients with PD in Germany had a higher number of amalgam fillings [7]. A retrospective cohort study in New Zealand evaluated the association between amalgam filling and the development of nervous system disorders, including PD [4]. The conclusion of limited evidence of an association between amalgam and diseases was made; however, the authors also discussed that cases were insufficient for the investigation of Alzheimer’s disease or PD. In the present study, 182 PD patients (n = 126 and 56 for the amalgam filling and control cohorts, respectively) were screened (Table 2).

**Table 4:** Previous published works comparing the outcomes, particularly the incidence rate of PD between amalgam filling patients (AF) and non-amalgam filling patients (non-AF).

Authors	Published year	Incidence rate (AF vs. non-AF)	Study Settings
Ngim CH *et al*.	1989	There was a clear monotonic dose-response association between PD and blood mercury levels.	A case-control study among the multiethnic population of Singapore to test the hypothesis that a high level of body burden mercury is associated with an increased risk of PD.
Seidler A *et al*.	1996	PD cases reported a higher number of amalgam fillings (n = 7.8) than both neighborhood controls (n = 6.5, *p* = 0.0008) and regional controls (n = 6.1, *p*<0.00005).	A case-control study comparing 380 German PD patients with 379 neighborhood controls and 376 regional controls
Bates MN *et al*.	2004	There were insufficient cases for investigation of the association between amalgam filling and PD.	A retrospective cohort study restricted to people who were at New Zealand Defense Force entry, were aged <26 years and had all their posterior teeth
Hsu YC *et al*.	Current study	Patients with dental amalgam filling(s) have a higher incidence of PD compared to those without dental amalgam filling(s) (adjusted HR: 1.583, *p*<0.01).	A population-based cross-sectional study in Taiwan enrolling 1,000,000 beneficiaries from the Longitudinal Health Insurance Database

The detailed reasons for the increased risk of PD in the amalgam filling cohort are still unknown; however, the elemental mercury (Hg^0^) in dental amalgam might play an important role. Elemental mercury (Hg^0^) in dental amalgam could convert to methylmercury (MeHg; CH_3_Hg^+^), which has a higher entry rate across the blood-brain barrier into the central nervous system (CNS) than inorganic mercury (Hg^+^ and Hg^2+^). Therefore, MeHg is posited as an important neurotoxicant [25]. The possible mechanisms of MeHg formation begin with the interaction of elemental mercury with other metals, creating a galvanic current, or the immersion of dental amalgam in sodium chloride or weak acids from foods. Both of the processes described cause the oxidative corrosion of amalgam, releasing inorganic mercury into the oral cavity. A positive correlation between the presence of organic mercury, presumably MeHg, in saliva and amalgam filling was previously demonstrated [26]. Moreover, inorganic mercury may be converted to MeHg by oral microorganisms [27]. High levels of MeHg in the CNS, resulting in oxidative stress, have been found to be a major factor in neurological diseases including dopamine- or glutamate-related apoptosis [28]. In addition, MeHg has a strong affinity for nucleophiles such as thiol- and selenol- molecules, whose roles are decisive for antioxidation and proper homeostasis of neuronal and glial cells [14, 29]. Therefore, as mentioned previously, the oxidative stress and interaction of MeHg with nucleophiles could induce neurotoxicity and neurodegenerative diseases such as PD.

In our present study, the amalgam filling cohort demonstrated a significantly higher incidence of potential comorbidities of PD, including diabetes, hypertension, hyperlipidemia, and cardiovascular disease than the cohort without amalgam filling (Table 1). As we know, impaired glucose regulation, hypertension, and dyslipidemia are main components of metabolic syndrome, which is a low-grade inflammatory condition associated with increased risk for diabetes and cardiovascular disease [30]. Studies have revealed that root caries is highly prevalent in patients with diabetes owing to periodontal attachment loss and gingival recession, compounded by reductions in salivary flow and elevated glucose level in their gingival crevicular fluid [31–35]. A study has also revealed that hyperglycemia and insulin resistance were in strong relation to dental caries [30]. Furthermore, amalgam was one of the commonly used filling materials in Taiwan [36]. In brief, metabolic syndrome or diseases might impair the normal physiological regulation, which may lead to dental caries and end up with amalgam filling. Therefore, we would expect a higher incidence of PD-related comorbidities in the amalgam filling cohort than in the non-amalgam filling cohort.

Among the amalgam filling cohort (Table 3), those with diabetes or hyperlipidemia had a decreased risk of PD and those with hypertension had an increased risk of PD. However, whether the above-mentioned comorbidities affect the risk of PD is controversial [37, 38]. Our present data demonstrate that having diabetes or hyperlipidemia had a protective effect against PD in the amalgam filling cohort. Metformin, a common oral antidiabetic agent, has been reported to possibly reduce the risk of dementia by decreasing advanced glycation end-products in the CNS [15] and acting on AMP kinase to restore more physiological cellular bioenergetics [39]. Being a neurodegenerative disease, PD has been reduced by administration of oral antidiabetic agents for type 2 diabetes as practiced in Taiwan in the period 1996–2007, and 84% of the patients used metformin [40]. Moreover, there is accumulating evidence that alterations in fat metabolism are involved in the pathogenesis of neurodegenerative diseases, including Alzheimer’s disease and PD [41]. Results from a previous study revealed an increased risk of PD in carriers of the *APOE* ε2 allele [42], which is associated with lower plasma levels of total cholesterol. However, this study does not present individual information on specific cholesterol fractions. On the other hand, hypertension had a reverse effect on PD among patients with amalgam filling. The biological mechanisms that link hypertension to PD are poorly understood and can only be speculative [43]. First, long-standing elevated blood pressure can result in ischemic cerebrovascular lesions, which are likely to lead to the clinical expression and deterioration of idiopathic PD symptomatology [44]. Second, oxidative stress and the renin-angiotensin system are common mechanisms that contribute to both PD and hypertension, which may mediate the association between the two diseases [45]. However, further investigations are needed to clarify the detailed role of amalgam filling in the association between PD and these comorbidities.

Certain limitations should be considered in the present study. First, the database cannot provide any other information about exposure to heavy metals, such as residential environment pollutants, occupation, and dietary habits, which are the hypothetic factors of PD. Furthermore, the lack of medical records from the database to evaluate the clinical conditions of the defined PD patients was due to ethical requirements. The aforementioned limitations might hamper the validation of the study. Therefore, to evaluate diagnostic accuracy, we examined the prevalence of PD in this cohort, which was comparable with that observed in the previous epidemiology study of PD in Taiwan [17]. Our cohort showed that the prevalence of PD per 1,000,000 people was 681 in 2003 and 1530 in 2008. This suggests that the diagnostic sensitivity of our study criteria is acceptable.

Second, the number of new amalgam fillings from 2000 to 2008 does not represent the actual number of fillings during the patients’ lifetimes. In the present study, the control group was not a real non-exposure group because these control individuals might have been exposed to amalgam fillings before year 2000. Therefore, the data obtained for the amalgam filling cohort might have resulted in an underestimation.

Third, the design of this study involved only a 7-year observation period, which is the major limitation of the study. Although the median survival time in this study was 5.74 years in the amalgam filling cohort, most of subjects were diagnosed with PD within 2 years after amalgam filling. It is unlikely that mercury would induce PD in such a short time. However, amalgam was one of the major filling materials used in Taiwan (53.3% of posterior tooth restorations) [36], and dental caries is considered a lifelong disease [46]. In addition, the Japanese-made light-cure composite resin (SHOFU DENTAL TAIWAN CO.) was first introduced in Taiwan in 1985 [47], and the filling material used before was mainly amalgam [36]. In the present study, therefore, the finding of patients having an amalgam filling within the observation period might also indicate that the patients were prone to have a history of dental caries and amalgam filling. The follow-up years for PD after exposure to amalgam filling might therefore be much less significant than the comparison of the incidences of PD between the 2 cohorts.

Finally, the risk of reverse causality bias might not be avoided. In other words, patients with prodromal and/or preclinical PD are more likely to ignore their healthcare and be at a higher risk of restorative procedures. However, the recent findings have revealed that the rates of visiting a dentist and receiving filling in the general population, including those with physical disabilities, increased because of the national health insurance policy in Taiwan [48]. Although the argument that patients with preclinical PD are more likely to ignore their healthcare might not be completely ruled out, the bias from the ignoring of healthcare might partly be reduced due to the national health insurance policy.

## Conclusions

The patients exposed to dental amalgam filling were 1.583 times more likely to develop PD afterward compared to their non-exposed counterparts, after adjusting for comorbidities and CCI scores. Owing to the limitations of the experiment, this does not imply that the patients were diagnosed with PD immediately after receiving amalgam filling. The present results could neither determine a causal relationship nor compare the time of onset of PD. Nevertheless, our findings showed that the elderly who received amalgam fillings had a higher risk of PD in the population-based 9-year retrospective cohort study in Taiwan. Future long-term follow-up studies are needed to examine environmental exposures, dietary habits, and accurately define PD diagnosis for better understanding of the association between dental amalgam and the development of PD in order to provide insight into the important clinical implications of this disease.
